# Antibiotic Resistance Genes in the Bacteriophage DNA Fraction of Environmental Samples

**DOI:** 10.1371/journal.pone.0017549

**Published:** 2011-03-03

**Authors:** Marta Colomer-Lluch, Juan Jofre, Maite Muniesa

**Affiliations:** Department of Microbiology, University of Barcelona, Barcelona, Spain; Cairo University, Egypt

## Abstract

Antibiotic resistance is an increasing global problem resulting from the pressure of antibiotic usage, greater mobility of the population, and industrialization. Many antibiotic resistance genes are believed to have originated in microorganisms in the environment, and to have been transferred to other bacteria through mobile genetic elements. Among others, β-lactam antibiotics show clinical efficacy and low toxicity, and they are thus widely used as antimicrobials. Resistance to β-lactam antibiotics is conferred by β-lactamase genes and penicillin-binding proteins, which are chromosomal- or plasmid-encoded, although there is little information available on the contribution of other mobile genetic elements, such as phages. This study is focused on three genes that confer resistance to β-lactam antibiotics, namely two β-lactamase genes (blaTEM and blaCTX-M9) and one encoding a penicillin-binding protein (mecA) in bacteriophage DNA isolated from environmental water samples. The three genes were quantified in the DNA isolated from bacteriophages collected from 30 urban sewage and river water samples, using quantitative PCR amplification. All three genes were detected in the DNA of phages from all the samples tested, in some cases reaching 104 gene copies (GC) of blaTEM or 102 GC of blaCTX-M and mecA. These values are consistent with the amount of fecal pollution in the sample, except for mecA, which showed a higher number of copies in river water samples than in urban sewage. The bla genes from phage DNA were transferred by electroporation to sensitive host bacteria, which became resistant to ampicillin. blaTEM and blaCTX were detected in the DNA of the resistant clones after transfection. This study indicates that phages are reservoirs of resistance genes in the environment.

## Introduction

Recognized as a global problem [1], antibiotic resistance increases the morbidity and mortality caused by bacterial infections, as well as the cost of treating infectious diseases. The threat from resistance (particularly multiple resistance in bacterial strains that are widely disseminated) is serious. The key factors contributing to this threat are the pressure of increased antibiotic usage (in both human and animal medicine), greater mobility of the population and industrialization [2], [3]. Many potentially life-threatening infections, generally regarded as diseases from the past due to the success of antibiotics and vaccines, have returned as resistance increasingly hampers successful therapy and prophylaxis [4].

Microorganisms produce many antimicrobials in nature [5], [6]. These antibiotic-producing organisms have also become resistant to the antibiotics they produce, and the genes that confer such resistance can be transferred to other non-resistant bacteria. The presence of antibiotics in the environment may provide long-term selective pressure for the emergence and transmission of these resistance-conferring genes in non-producing organisms [5], [7]. Given that many genera found in diverse environments carry resistance determinants [6], it is feasible that antibiotic-resistance genes have originated in the environment and that they could have been transferred from the environment to pathogenic bacteria, which are currently found in clinical settings. [8]. The transfer from the environment to clinical settings might have occurred through horizontal gene transfer, which is the most effective mechanism to accelerate the dispersal of antibiotic-resistance genes. The mobile genetic elements (MGEs) for the horizontal transfer of such genes most commonly studied are plasmids, transposons or, as a few reports suggest, bacteriophages [9]–[11].

Several studies have focused on antibiotic resistance codification in plasmids or transposons, and there is also interesting information about the extent of antibiotic resistance genes in a given environment (the so-called “resistome”) [8], [12]. However, there is less information on the potential contribution of phages to antibiotic resistance-gene transfer, despite calls for research in this field. Recent reports [2], [11] conclude that the horizontal transfer of genetic information by phages is much more prevalent than previously thought, and that the environment plays a crucial role in the phage-mediated transfer of antibiotic-resistance genes [2], [13]. Since many antibiotic resistance genes are plasmid-encoded, much effort has been devoted to the study of plasmids and less to the study of phages carrying genes for antibiotic resistance. However, many reports available suggest that phages can mobilize resistance genes and confer resistance, and some authors suggest that mobilization can occur through generalized transduction [14]–[18]. Only a few reports have analyzed antibiotic resistance genes in phage DNA isolated from wastewater environments [9], [19].

β-lactam antibiotics are characterized by clinical efficacy and low toxicity and they are thus widely used as antimicrobials. One mechanism of resistance to β-lactam antibiotics in Gram-negative bacilli involves the production of β-lactamases [3]. Among other Gram-negative bacteria, members of the family *Enterobacteriaceae* commonly express plasmid-encoded β-lactamases (e.g. TEM/SHV), which confer resistance to penicillins. More recently, extended-spectrum β-lactamases (ESBLs) evolved, conferring resistance to penicillins and oxymino-cephalosporins. EBSLs are sometimes mutant derivatives of TEM/SHV, but they are also mobilized from environmental bacteria (e.g. CTX-M) [20]. Most β-lactamases are acquired by horizontal gene transfer and the novel β-lactamase genes that emerge dramatically spread worldwide, causing both nosocomial and community-onset infections [3].

Resistance in Gram-positive bacteria is also widely distributed and increasing. This is the case for the emergence of community-associated methicillin-resistant *Staphylococcus aureus* (MRSA), a development that has blurred the distinction between hospital and community strains [21]. In *S. aureus, mec*A, a gene encoding for a penicillin-binding protein that confers resistance to methicillin, is located on a mobile genomic island, the Staphylococcal Cassette Chromosome mec (SCCmec) [22], [23]. In addition to the resistance genes carried on SCC*mec*, *S. aureus* can also harbor resistance genes on other sites of the genome, such as Tn*554*, as well as on plasmids [23]. Antibiotic use and environmental factors contribute to the emergence and spread of resistance in *S. aureus*, which is a common cause of serious and life-threatening infections.

Here we focused on two β-lactamases (*bla*_TEM_ and *bla*_CTX-M_) and a penicillin-binding protein (*mec*A). *bla*_TEM_ belongs to class A serine β-lactamases, which have been described in epidemiological studies; *bla*_CTX-M_ and *bla*_TEM_ are the most prevalent broad-spectrum β-lactamases and the most widely distributed enzymes worldwide [24]–[26]. *mec*A was included in this study because of the increasing incidence of infections caused by MRSA. The three genes were quantified by real-time PCR in the viral DNA fraction of water samples contaminated with fecal pollution. Since in most environments studied, phages are the main part of the viral fraction [27], it can be assumed that the DNA isolated from the viral fraction will belong mostly to bacteriophages. We sought to highlight the potential role of phages in the spread of these genes in the aquatic environment.

## Results

### Microbiological parameters

The numbers of aerobic bacteria and *Escherichia coli* were relatively homogeneous in all the urban sewage and river water samples tested (Table 1). These values were in accordance with previous water analyses from the same source [28]–[30]. River water samples showed significantly lower numbers (*P*<0.05) than urban sewage and these differences are attributed to the lower fecal input received by river water. The numbers of resistant bacteria were slightly lower than the total bacteria, as expected. Since bacteria are difficult to recover from the environment because of the stressed conditions of bacterial cells, the method and the low concentration of ampicillin (35 mg/ml) used were intended to prevent the inhibition of growth. Similar concentrations of ampicillin were reported before for the isolation of ampicillin-resistant bacteria [31]. We further tested 10% of all the colonies isolated in LB agar plates (35 mg/l) for sensitivity at higher concentrations of ampicillin (100 mg/l). At this concentration all the isolates were resistant to the antibiotic.

**Table 1:** Samples analyzed and microbiological parameters.

Sample	Urban sewage		River	
	Average log_10_ CFU/ml	SD	Average log_10_ CFU/ml	SD
N	15		15	
Aerobic bacteria	6.47	0.32	3.71	0.37
*E. coli*	4.75	0.64	1.22	0.56
*S. aureus*	2.29	0.36	1.88	0.11
Aerobic bacteria ap^R^	6.22	0.24	3.12	0.45
*E. coli* ap^R^	4.14	0.34	0.80	0.56
*S. aureus* met^R^	1.51	0.20	0.00	-
Somatic coliphages^a^	4.43	0.30	2.42	0.39

a PFU/ml.

To determine the number of *S. aureus* strains in the samples, 25% of the yellow-pigmented colonies obtained in each plate of agar 110 medium were further confirmed by catalase and with the Slidex Staph Plus kit. Depending on the plates, from 80% to 90% of the colonies were confirmed as *S. aureus.* The numbers of these bacteria presented (Table 1) are a correction of the percentage of positive colonies among the total number of yellow colonies detected in the agar plate. We detected S. *aureus* MRSA in sewage but not in river water.

Somatic coliphages, proposed as viral fecal indicators of pollution [29], were analyzed to determine the presence of bacteriophages infecting *E. coli* in the samples studied. As for bacterial indicators, the numbers of somatic coliphages were relatively homogeneous in all the samples tested (Table 1) and also in accordance with previous analyses of samples from the same source [28]–[30].

### Direct observation of bacteriophages in sewage and river water

**Figure 1:**
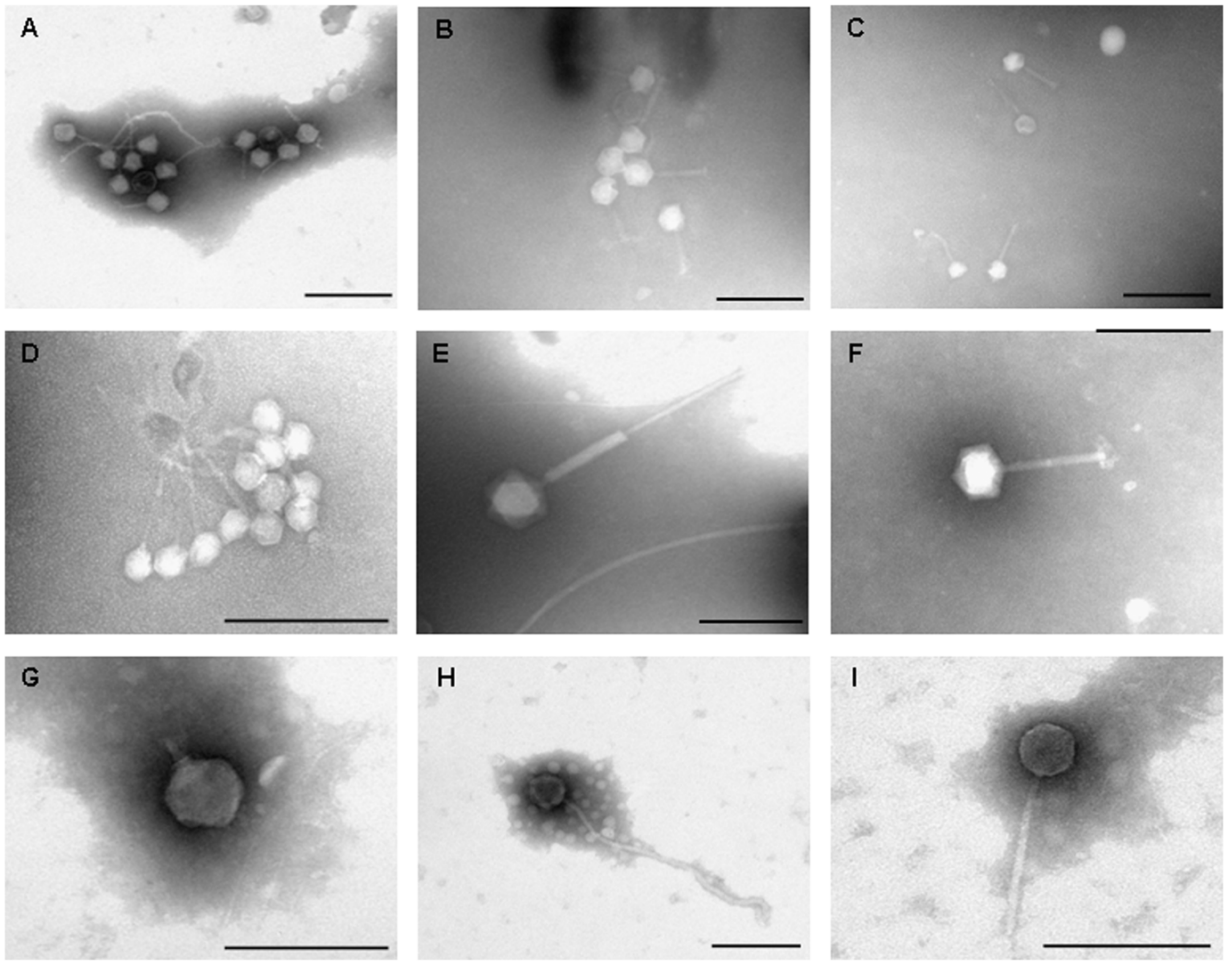
Electron micrographs of bacteriophages present in sewage and river water. A–B. Group of phages with *Myoviridae* and *Siphoviridae* morphology from sewage. C. *Myoviridae* phages from river water. D: group of *Siphoviridae* phages from sewage. E–F. *Myoviridae* phages from sewage. G: *Podoviridae* phage from sewage. H–I. *Siphoviridae* phages from sewage and river water respectively. Bar 200 nm.

In addition to the evaluation of infectious somatic coliphages in the samples, direct observation of bacteriophages present in the water samples was conducted by electron microscopy. Tailed bacteriophages (Figure 1) belonging to different morphological types were observed, with a greater abundance of phages with contractile tail with *Myoviridae* morphology and non-contractile tail with *Siphoviridae* morphology. Variations in capsid and tail size were observed, as expected for bacteriophages that can infect different bacterial genera. Non-tailed virus particles were also observed, although in this case it could not be determined by morphology whether they were bacterial viruses or viruses infecting other hosts.

### Antibiotic resistance genes in the phage and bacterial fraction of sewage and river water *bla*_TEM_ genes

**Figure 2:**
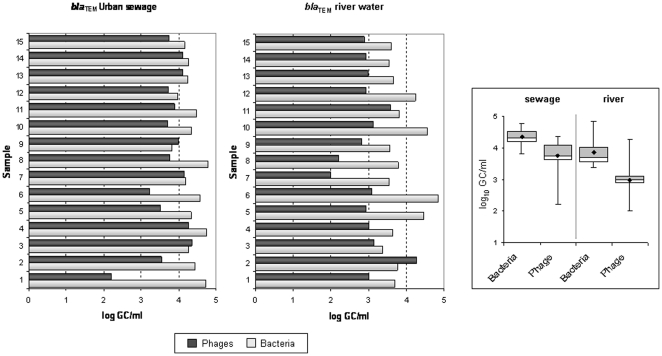
Number of copies of *bla*_TEM_ genes (GC/ml) in urban sewage and river water samples in phage and bacterial DNA. On the left side of the figure, bar chart of the gene copies detected for each sample, dark grey for phage DNA and light grey for bacterial DNA. On the right side of the figure, the box plot chart shows the averaged values obtained from all samples from the same origin. Within the box plot chart, the cross-pieces of each box plot represent (from top to bottom) maximum, upper-quartile, median (black bar), lower-quartile, and minimum values. Black diamond shows the mean value. The grey boxes in the box plot chart include samples showing values within the 75th percentile and white boxes samples showing values within the within the 25th percentile.

The set of primers and probe used [25], which included amplification of more than 145 TEM variants, allowed efficient screening of *bla*_TEM_ genes in the environmental samples. From 10^2^ to10^4^ *bla*_TEM_ gene copies (GC) were detected in the phage DNA fraction of one ml of urban sewage (Figure 2), while in river water the average was one order of magnitude lower. In both types of sample, these values indicate that phage DNA contains a large number of *bla*_TEM_ gene copies. As explained in the methods section, a careful approach was performed to rule out that DNA from a non-viral origin was amplified in the qPCR, and controls were performed during phage DNA extraction. To this end, controls of the samples, taken after DNase treatment, but before the phage DNA was extracted from the capsid, were used as template for conventional PCR for eubacterial 16S rDNA and for qPCR for the three antibiotic resistance genes. These controls showed negative values for eubacterial 16SrDNA as well as for the three antibiotic resistance genes, which confirmed that the samples were free of bacterial DNA or non-encapsidated DNA, and that our results were due to amplification of DNA located within the viral particles. These controls were performed in all the samples tested.

The number of copies of *bla*_TEM_ genes detected in the phage DNA fraction of the samples were, as expected, lower than in bacterial DNA; however, this difference was less than one order of magnitude (Figure 2). Differences in GC/ml found between bacterial and phage DNA were significant (*P*<0.05). Moreover, a few samples (Samples 3 and 9 in sewage and Sample 2 in river water, Figure 2) showed a higher concentration of *bla*_TEM_ in phage DNA than in bacterial DNA.

### *bla*_CTX-M_ genes

**Figure 3:**
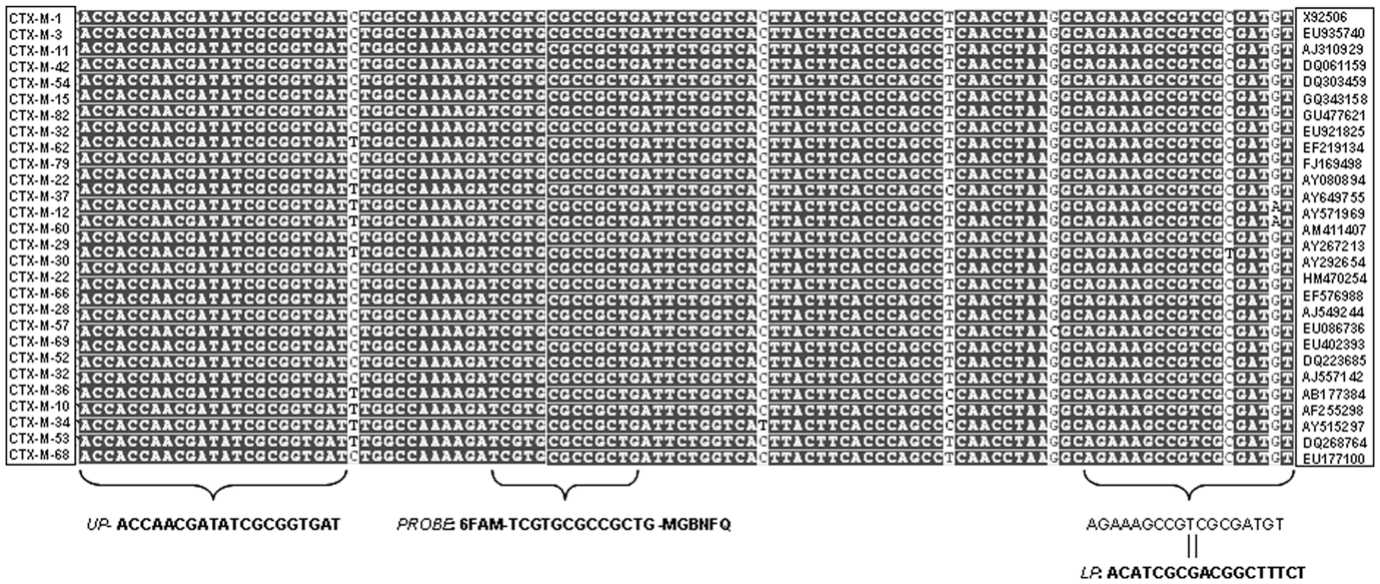
Primers and TaqMan assay probe resulting from the alignment of *bla*_CTX-M_ genes from Cluster 1. Conserved nucleotides are marked in bold, non-conserved nucleotides in white. Sequence reverse and complementary is shown for lower primer. Right column indicate the GenBank accession number of each gene.

To our knowledge, quantitative real-time PCR probes that are universal for the most common variations of *bla*_CTX-M_ genes have not previously been reported, and so a primer set for these genes was developed in this study. The nucleotide sequence for diverse *bla*_CTX-M_ genes was aligned in a search for common sequences. As expected, the five clusters described for the CTX-M family did not share conserved regions (see references [32], [33] for review and presentation of a CTX-M cluster), so it was impossible to design a common qPCR for all the CTX-M variants. We selected Cluster 1 (composed of 31 variants described so far, including CTX-M-1, 3, 10, 11 and 15) [34], which is widespread in Europe and Spain [25], [35]. Alignment of some CTX-M Cluster 1 sequences (Figure 3) showed several regions from which primers and probe can be selected according to the requirements for the design of primers and probes for qPCR, established in the Primer Express Software version 3.0 (Applied Biosystems). The Taqman PCR assay developed was valid for quantitative measurements of all Cluster 1 CTX-M variants assayed, except CTX-M-12, 30 and 60, which did not match the sequence of the lower primer (Figure 3). Standard curves were repeatable and the amplification efficiency (*E*) of our reactions ranged from 95%-100%. Controls performed with several *E. coli* strains harboring different CTX-M genes from Cluster 1 confirmed the validity of the qPCR set designed.

**Figure 4:**
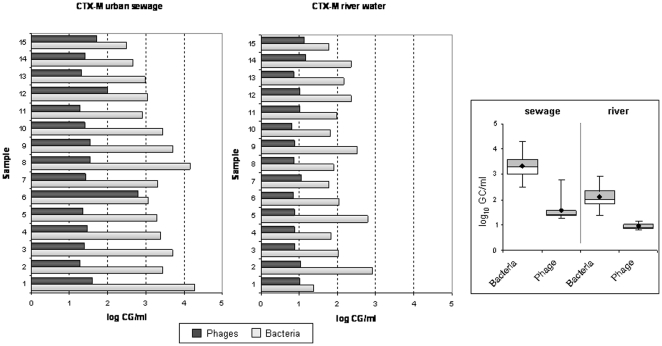
Number of copies of *bla*_CTX-M_ genes (GC/ml) in urban sewage and river water samples in phage and bacterial DNA and box plot of averaged values.

The number of copies of *bla*_CTX-M_ detected in phage DNA in sewage ranged from 1.5 to 3 log_10_ units, while fewer than one log_10_ units were still detected in one ml of river water (Figure 4). Differences between the number of copies of the *bla*_CTX-M_ genes in phage and bacterial DNA were significant (*P*<0.05) in both sewage and river water. The number of copies of the gene detected in phage DNA were from <1 to 2.5 log_10_ units lower than in bacterial DNA, with few exceptions (sewage sample 6). The number of copies of the *bla*_CTX-M_ genes in bacterial DNA was as high as 4 log_10_ units in 1 ml of sewage and almost 3 log_10_ units in 1 ml of river water.

### mecA

**Figure 5:**
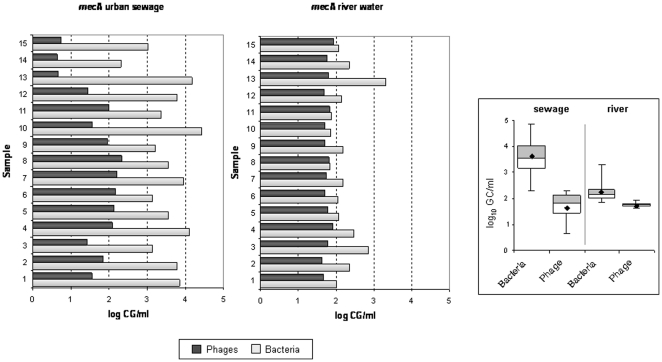
Number of copies of *mec*A (GC/ml) in urban sewage and river water samples in phage and bacterial DNA and box plot of averaged values.

All samples showed the presence of *mec*A in either bacterial or phage DNA. While values in bacterial DNA were higher in sewage, in phage DNA the average and also inter-sample comparison showed that some samples of sewage presented lower values than river water samples. The variability of the *mec*A content in phage and bacterial DNA in urban sewage samples was greater than in river samples (Figure 5).

### Ability of phage-encoded genes to confer antibiotic resistance in bacterial strains

To evaluate whether the antibiotic resistance sequences in phage DNA correspond to potential active genes able to confer resistance in a bacterial background, phage DNA from sewage samples 6, 7 and 15, was transfected in two *E. coli* recipient hosts (C600nal^R^ and WG5), both of which nalidixic acid-resistant and ampicillin-sensitive. After transfection, *E. coli* colonies were selectively grown in Chromocult ap/nal plates (Table 2). 25% of the ap/nal resistant *E. coli* colonies in each plate were randomly selected and analyzed for *bla* genes using conventional PCR with the respective primers (Table 2) and confirmed by sequencing. More ap-resistant clones were detected using WG5 as recipient than C600nal^R^. Analysis of the *bla* genes located in each clone showed from 0–10% of the clones harbouring *bla*_TEM_ or *bla*_CTX-M_. Among these, more clones harboring *bla*_TEM_ and *bla*_CTX-M_ were also found with WG5. *bla*_TEM_ was detected in a greater percentage of colonies than *bla*_CTX-M_ in both host strains and no clones were detected for *bla*_CTX-M_ in C600nal^R^ on two of the three samples assayed (Table 2). Accordingly, the densities of *bla*_TEM_ genes in the sewage samples used were greater than densities of *bla*_CTX-M_ (Figs. 2 and 4 respectively). Both genes were never detected simultaneously in a single clone. Other clones showing nal/ap resistance were not harboring the two *bla* genes analyzed, suggesting that other gene conferring ampicillin resistance could have been transferred.

**Table 2:** Transfection of phage DNA isolated from sewage in *E. coli* WG5 and C600 strains.

		Sample number		
		Sewage 6	Sewage 7	Sewage 15
	µg of phage DNA transfected	2.60	1.14	1.74
Ampicillin/Nal WG5	N° of ap/nal resistant clones^a^	552	422	310
	% *bla*_TEM_ b	10.0	13.6	16.6
	%*bla*_CTX-M_ b	6.8	1.7	13.3
Ampicillin/Nal C600nal^R^	N° of ap/nal resistant clones^a^	89	101	42
	% *bla*_TEM_	3.6	7.7	6.2
	%*bla*_CTX-M_	0	1.2	0

a Averaged number of colonies per plate after transduction.

b Percentage of colonies where these genes have been detected by PCR and confirmed by sequencing.

## Discussion

Genes of antibiotic resistance are present in bacterial chromosomes and they are detected in plasmids when analyzed in clinical settings, but there is controversy as to how these genes originate and how they reach the pathogenic strains found in hospitals. Several authors indicate a plausible environmental origin of these genes, and we suggest here that phages could be suitable candidates as intermediates between the original bacteria and the clinical isolate.

The genes examined in the present study are the most widely distributed. TEM has been reported worldwide [36] and CTX-M is currently the most widespread and threatening mechanism of antibiotic resistance, particularly in community-acquired infections [25]. The qPCR set designed for CTX-M detected one of the five main clusters described for *bla*_CTX-M_ genes [20]. Cluster 1 is one of the most diversified groups, which is of particular interest because of the recently described international spread and changing epidemiology of clones carrying the CTX-M-15 variant [3], [5], [34], [37]. Although the qPCR set detected other types in addition to type 15, the prevalence of *bla*_CTX-M-1_ in phage DNA (Figure 4) indicates this cluster is abundant in environmental phage DNA. Our results may be applicable to other CTX-M clusters, and it is feasible that the other clusters would also be detectable in phage DNA. Recent studies suggest that the CTX-M-type derives from chromosomal genes from several *Kluyvera* species and that it is rapidly mobilized from these species to a number of genetic platforms [20], such as insertion sequences, integrons, transposons and plasmids.

We detected MRSA in sewage. although in other studies *Staphylococcus* was not detected in municipal wastewater [38], or it was detected but not quantified [39]. The results of *mec*A in phage DNA showed a lack of correlation with fecal pollution in the samples, since averaged values of sewage and river water were similar. This suggests that the *mec*A detected came from phages other than those found in human fecal pollution. Although the sewage samples analyzed contain exclusively human fecal pollution river samples in this study carried mostly human fecal pollution but also some animal fecal pollution [29], as well as autochthonous freshwater bacteria. Since previous experiments with these urban sewage samples indicated that the values of fecal pollutants are highly consistent over time [30], the variability in the number of copies of the *mec*A detected in phages supports the hypothesis of an origin other than the human fecal load. Our results do not allow us to discern whether the gene derives from animals or autochthonous microorganisms.

*S. aureus* can mobilize fragments of its chromosome, the pathogenicity islands, or with helper phages [40]. The transfer of *S. aureus* phages into and out of isolates may occur in nature or during the course of colonization or infection of patients [23]. The number of copies of *mecA* detected in phage DNA supports our hypothesis that, regardless of its origin, *mec*A is located in phages in aquatic environments. This wide spread of *mec*A could have influenced the emergence of community-acquired strains, which are responsible for serious diseases in healthy individuals [41].

The occurrence of antibiotic resistance genes in the viral DNA fraction of water samples provides new insights into the extent to which ecosystems serve as pools of resistance genes and suggests that phage DNA can act as reservoirs of these genes. However, our results do not indicate whether these genes confer resistance in a given bacterial host. To elucidate this point, a set of experiments aiming to transduce the genes from phage particles isolated from the samples in *E. coli* was attempted. Unfortunately, as shown in other studies [42], this approach might need to identify a suitable and sensitive host strain (*E. coli* or others) that would support infection with these phages and subsequent transduction. The search for the suitable host and the right conditions for transduction to occur is likely a complicated task. Moreover, the phages in which antibiotic resistance genes were detected are not necessarily infectious particles. We were therefore unable to achieve transduction of the antibiotic resistance to a bacterial host strain (data not shown), although more efforts will be made to pursue this objective.

However, we were able to demonstrate that the sequences corresponding to resistance genes detected in phage DNA can confer resistance to a recipient bacteria. Using *E. coli* as a Gram-negative host we generated resistant clones after transfection of phage DNA. This approach avoids the requirement of a suitable host strain and the need for phage infectivity, and only requires a suitable genetic background in which the genes can be expressed. The *bla*_TEM_, *bla*_CTX-M_ genes were transferred into the host strains, which then became resistant to the respective antibiotics after transfection of environmental phage DNA. This demonstrates that these genes can be expressed in a bacterial genetic background.

A similar experimental approach was attempted with environmental phage DNA carrying *mec*A in an *S. aureus mec*A^−^ strain, although no methicillin-resistant colonies were obtained (data not shown). This is not surprising since methicillin resistance is conferred by acquisition of the SCC*mec* element, which includes a type-specific *ccr* complex, and the *mec* complex, which includes *mec*A and its regulatory genes [22], [43]. Although a complete SCC*mec* element may not be needed, at least a complete *mec* complex seems to be necessary for the expression of methicillin-resistance. *mec*A is always localized within *mec* complexes in all reported MRSA isolates and it is never transferred alone. The various SCCmec elements are between 21 and 67 kb, so it is unlikely that a phage would carry such a long, active SCC*mec* element, which could then be transferred and confer resistance.

Several reports relate wastewater and antibiotic resistance [39], [44], [45]. Many characteristics of wastewater make it a highly suspect medium for the spread of antibiotic resistance genes, i.e., the presence of antibacterials from household products (soaps, detergents, etc.), the presence of antibiotics that have been excreted by humans or disposed of down the drain, and a high bacterial load. The evolution of MGEs, which allow horizontal gene transfer, depends on the selective forces operating on them, independently of the host strain. However, these elements often encode products with a selective value for the host, and bacteria increase their fitness and diversity when they acquire these elements. In this case, the incorporation of antibiotic resistance in environments with high antibacterial concentrations would guarantee the survival of the bacterial host.

There are only a few examples of antibiotic resistance genes identified as elements of phage chromosomes. However, phages mobilize antibiotic resistance genes through generalized transduction, as reported in several bacterial genera [14]–[16]. Other “phage-like particles” may also be responsible for the spread of antibiotic resistance genes [46]. *In vitro*, phages transduce resistance to imipenem, aztreonam and ceftazidime in *Pseudomonas aeruginosa* by generalized transduction [12]. The epidemic strain *Salmonella enterica* serovar Typhimurium DT104, characterized by various multiresistance patterns, transduces some of the resistance genes [14]. *Bacillus anthracis* temperate phage encodes demonstrable fosfomycin resistance [47]. Since 1970s evidence has been presented that prophages participate in the dissemination of erythromycin-resistance phenotype *Streptococcus* infections [17], [48]. The *mefA* gene, encoding macrolide resistance, is associated with a 58.8-kb chimeric genetic element composed of a transposon inserted into a prophage in *S. pyogenes* [18]. The *ermA* gene, a erythromycin resistance determinant, is located on an integrated conjugative element present in Streptococcus strain GAS [49]. These mobile elements identified for macrolide transfer can contribute to mobilization of the genes studied here. However experimental identification of the resistance determinant within a phage is needed.

Other indirect evidence for beta-lactam antibiotics mobilized by phages has been reported. CTX-M-10 was linked to a phage-related element which disseminates among *Enterobacteriacea* in a hospital [46]. We agree with these authors that the transfer of *bla*_CTX-M-10_ from the chromosome of *Kluyvera* spp. to a transferable plasmid may have been mediated by transduction by a phage. Genetic analyses of *Kluyvera* phages revealed high homology with phages infecting *E. coli* [50]. This observation indicates that recombination between the two phages facilitated gene exchange between these bacterial genera. In 1972, Smith [51] reported ampicillin resistance conferred by phage infection, but these studies were not pursued. We previously described the presence of phages encoding sequences of *bla*_OXA-2_, *bla*_PSE-1_ or *bla*_PSE-4_ *and bla*_PSE_-type genes in sewage. This was the first report of the contribution of phages to the spread of β-lactamase genes in the environment [9], although the genes detected were not quantified.

Phages, either lytic or temperate, usually persist better in water environments than their bacterial hosts do [28], [29]. This higher survival makes them suitable candidates for transferring genes among bacteria. Due to the structural characteristics of phages, their persistence in the environment is higher than free DNA (either linear fragments or plasmids), which is more sensitive to nucleases, temperature, predation and radiation [52]–[54]. This observation supports the notion that the contribution of phages to gene transfer in natural extra-intestinal environments and in human-generated environments is greater than that of plasmids or transposons. Plasmids and transposons may be the main routes for antibiotic resistance transfer In clinical settings. However, the fact that they are degraded faster than phages limits their role as MGEs in the environment.

The presents study shows that phages carry antibiotic resistance genes able to confer resistance to a bacterial strain. The possibility of transfer of these genes that lead to the emergence of new clones will depend on the susceptibility of infection of the recipient strains as well as the environmental conditions, but it could be assumed that it is likely to occur, although probably at a low frequency. In-depth analysis of the environmental dissemination of phages carrying antibiotic resistance genes outside the clinical setting could increase information about the antibiotic resistance genes circulating among the healthy human population, and their influence on the generation of resistance in the environment. Antibiotic resistance will continue to develop more rapidly than the new antimicrobial agents generated to treat infections, and mobilization through MGEs ensures dissemination of these genes. In many examples, the presence of antibiotics will increase SOS responses, which allows the mobilization of MGEs carrying antibiotic resistance genes, thereby ensuring their own dissemination [55]. It is, therefore, crucial to determine the mechanisms behind the spread of antibiotic resistance genes and to identify the new genes before they become a public health problem.

## Materials and Methods

### Bacterial strains, bacteriophages and media

*E. coli* strain C600 containing pGEM vector was used as a control for *bla*_TEM_. *E. coli* strains isolated from sewage during this study were used as controls for *bla*_CTX-M_ genes carrying types CTX-M 1, 3, 10, 11, 15 and 34. *S. aureus* MRSA isolated from a human patient was used as a positive control for *mec*A. *E. coli* strain WG5 (a nalidixic acid-resistant mutant) (ATCC 700078) (anonymous) and strain C600nal^R^ [56] were used as host for transfection experiments.

Luria-Bertani (LB) agar or broth was used for routine bacterial propagation. Chromocult® Coliform Agar (Merck, Darmstadt, Germany) and Staphylococcus Medium 110 (Difco Laboratories, France) were used to evaluate background flora. When necessary, media were supplemented with ampicillin (35 mg/l or 100 mg/l), 10 mg/l methicillin, or nalidixic acid (25 mg/l) (Sigma-Aldrich, Steinheim, Germany).

### Samples

#### Urban sewage

We used 15 sewage samples collected from the influent of an urban sewage plant that serves the urban area of Barcelona, including a number of cities and towns, of approximately 500 000 inhabitants. Samples were collected regularly approximately every 15 days over six months.

#### River sample

Fifteen samples were collected from the Llobregat river, near Barcelona, a watercourse that receives mixed human and animal contamination. Samples were collected regularly approximately every 15 days over six months.

### Microbiological parameters

Aerobic bacteria present in the samples and grown in TSA were evaluated by performing decimal dilutions of the sample in PBS, plating 0.1 ml of each dilution in TSA and incubating plates in aerobic conditions at 37°C for 18 h. *E. coli* was detected using Chromocult as an indicator of bacterial fecal pollution by the membrane filtration method, as described elsewhere [57]. Somatic coliphages, proposed as indicators of viral fecal pollution [58], were enumerated using the ISO method [59]. The estimation of total bacteria and *E. coli* resistant to β-lactam antibiotics was performed as described above but using TSA and Chromocult respectively supplemented with 35 mg/l of ampicillin.

Estimation of *S. aureus* in the same samples was done with Staphylococcus Medium 110 (Difco Laboratories, France), which was incubated at 37°C for 48 h for the isolation of staphylococci. For the estimation of methicillin-resistant *S. aureus*, agar plates supplemented with 10 mg/l methicillin (Sigma-Aldrich. Spain) were used. Colonies grown in this medium that showed yellow-orange pigment were suspected of being *S. aureus.* This was confirmed with the Slidex Staph Plus (Biomerieux España, Madrid. Spain).

### Standard PCR procedures

PCRs were performed with a GeneAmp PCR system 2700 (Applied Biosystems, Barcelona, Spain). The DNA template was prepared directly from two colonies of each strain suspended in 50 µl of double-distilled water and heated to 96°C for 10 min prior to the addition of the reaction mixture. Purified bacterial or phage DNA was diluted 1:20 in double-distilled water. The oligonucleotides used to amplify *mec*A, *bla*_TEM_ or *bla*_CTX-M_ are described in Table 3. Five µl of each PCR product was analyzed by agarose (1.5%) gel electrophoresis and bands were visualized by ethidium bromide staining. When necessary, PCR products were purified using a PCR Purification Kit (Qiagen Inc., Valencia, USA).

**Table 3:** Oligonucleotides used in this study.

Target gene	PCR	Sequence	Conditions	Amplimer (bp)	Reference
16SrDNA	UP	AAGAGTTTGATCCTGGCTCAG	95°C 5 min (1 cycle); 95°C 1 min, 42°C 0.5 min, and 72°C 2 min (35 cycles), 72°C 2 min (1 cycle).	1503	[61]
	LP	TACGGCTACCTTGTTACGACTT			
TEM PCR	UP	CTCACCCAGAAACGCTGGTG	95°C 5 min (1 cycle). 94°C, 15 s, 63°C 1 min, 72°C, 1.3 min (30 cycles). 72°C, 4 min (1 cycle).	569	This study
	LP	ATCCGCCTCCATCCAGTCTA			
TEM qPCR	UP	CACTATTCTCAGAATGACTTGGT	50°C 2 min (1 cycle). 95°C 15 min (1 cycle) 94°C for 15 s and 60°C 1 min (45 cycles).	85	[36]
	LP	TGCATAATTCTCTTACTGTCATG			
	Probe	6FAM-CCAGTCACAGAAAAGCATCTTACGG-MGBNFQ			
CTX-M-1 PCR	UP	ACGTTAAACACCGCCATTCC	95°C 5 min (1 cycle). 94°C, 15 s, 60°C 1 min, 72°C, 1.3 min (30 cycles) 72°C, 4 min (1 cycle).	356	This study
	LP	TCGGTGACGATTTTAGCCGC			
CTX-M-1 qPCR	UP CTX-M	ACCAACGATATCGCGGTGAT	50°C 2 min (1 cycle). 95°C 15 min (1 cycle) 94°C for 15 s and 60°C 1 min (45 cycles).	101	This study
	LP CTX-M	ACATCGCGACGGCTTTCT			
	Probe	6FAM – TCGTGCGCCGCTG- MGBNFQ			
MecA PCR	UP	ATACTTAGTTCTTTAGCGAT	95°C 5 min (1 cycle). 94°C, 15 s; 48°C 1 min, 72°C, 1.3 min (30 cycles). 72°C, 4 min (1 cycle).	434	This study
	LP	GATAGCAGTTATATTTCTA			
MecA qPCR	UP	CGCAACGTTCAATTTAATTTTGTTAA	50°C 2 min (1 cycle). 95°C 10 min (1 cycle). 95°C for 15 s and 60°C 1 min (40 cycles)	92	[38]
	LP	TGGTCTTTCTGCATTCCTGGA			
	Probe	FAM-AATGACGCTATGATCCCAATCTAACTTCCACA-TAMRA			

### qPCR procedures

#### Preparation of standard curves

For the generation of standards for the qPCR assays, a plasmid construct was used. The 569-bp fragment of TEM, the 356-bp fragment of CTX-M, and the 434-bp fragment of *mec*A, all obtained by conventional PCR with the primers described in Table 3, and purified as described above, were cloned with a pGEM-T Easy vector for insertion of PCR products, following the manufacturer's instructions (Promega, Barcelona, Spain). The construct was transformed by electroporation into *E. coli* DH5α electrocompetent cells. Cells were electroporated at 2.5 kV, 25 F capacitance and 200 Ω resistance.

Colonies containing the vector were screened by conventional PCR to evaluate the presence of the vector containing each insert. The presence of the insert in the vector and its orientation was assessed by conventional PCR and sequencing, as described above, using the primers in Table 3. The vector containing the insert was purified from the positive colonies using the Qiagen Plasmid Midi purification kit (Qiagen Inc., Valencia, CA, USA) and the concentration of the vector was quantified by a NanoDrop ND-1000 spectrophotometer (NanoDrop Technologies. Thermoscientifics. Wilmington. USA). The reaction product was linearized by digestion with *Xmn*I restriction endonuclease (Promega Co., Madison, USA). The restricted product was purified and quantified again.

To calculate the number of construct gene copies (GC), the following formula was used: [concentration of the pGEM-T- Easy::*insert* (ng/µl)/molecular weight (ng/mol)] ×6.022×10^23^ molecules/mol  =  n° molecules pGEM-T-Easy::*insert*/µl. The number of GC/µl of the stock prepared for each gene was calculated. Serial decimal dilutions of this stock were made in double-distilled water to prepare the standard curve for qPCR. The standard dilutions were then aliquoted and stored at −80°C until use. Three replicates of each dilution were added to each qPCR reaction.

#### blaCTX-M primers and probe set

Using the software tool Primer Express 3.0 (Applied Biosystems), primers and probes were selected for use in a standardized TaqMan amplification protocol. All primers and FAM-labeled fluorogenic probes were commercially synthesized by Applied Biosystems (Spain). CTX-M probe was a Minor groove binding probe with a FAM reporter (FAM: 6-carboxyfluorescein) and a non-fluorescent quencher (NFQ). Primers and probes were used under standard conditions in a Step One Real Time PCR System (Applied Biosystems, Spain). Primer and probe specificity was determined with sequence alignments using BLAST and NCBI data entries. The primers and probe set was tested for cross-reactions with the respective sensitive strains. Amplification was performed in a 20 µl reaction mixture with the TaqMan Environmental Real Time PCR Master Mix 2.0 (Applied Biosystems, Spain). The mixture contained 2 µl of the DNA sample or quantified plasmid DNA. Thermal cycler conditions were as follows: an initial setup of 10 min at 95°C, and forty cycles of 15 s of denaturation at 95°C, and 1 min of annealing/extension at 60°C. All samples were run in triplicate, as well as the standards, and positive and negative controls. The number of GC was defined as the average of the triplicate data obtained.

To screen for PCR inhibition, dilutions of the standard were spiked with environmental DNA and the experimental difference was compared to the true copies of the target genes in the standards. Inhibition of the PCR by environmental DNA was not detected.

### Purification of phage DNA

Fifty ml of sewage and 100 ml of river water samples were passed through low protein-binding 0.22-µm-pore-size membrane filters (Millex-GP, Millipore, Bedford, MA). When necessary, several filter units were used to filter the whole volume. This allowed us to partially purify viral particles from the samples- The viruses were then 100-fold concentrated by means of protein concentrators (100 kDa Amicon Ultra centrifugal filter units, Millipore, Bedford, MA), following the manufacturer's instructions. The total volume was reduced to 0.5 ml. The centrifugation time varied depending on the sample and ranged from 10–90 min. The viral concentrate was recovered from the tube and the volume was adjusted to 2 ml with double distilled sterile water. Samples were treated with DNase (100 units/ml of the viral concentrate) to eliminate free DNA outside the phage particles.

#### Control of non-phage DNA

An aliquot of the sample at this stage was evaluated to rule out the presence of bacterial or non-encapsidated DNA. After DNase treatment, but before desencapsidation, the samples were used as template for conventional PCR of eubacterial 16SrDNA (Table 3) and for qPCR of the three antibiotic resistance genes (Table 3). This control was to ensure that the DNase treatment had removed all the non-encapsidated DNA from the samples.

DNA from the viral particles was isolated by proteinase K digestion and phenol/chloroform (1∶1) (v∶v) treatment [59]. The mixture phenol/chloroform/phage lysate was added to Phase Lock Gel tubes (5- Prime, VWR International, Madrid, Spain) and centrifuged following the manufacturer's instructions. The DNA from the supernatant was precipitated using 100% ethanol and 3M sodium acetate, and the volume was adjusted to 250 µl. DNA was further purified by using Microcon YM-100 centrifugal filter units (Millipore, Bedford, MA), following the manufacturer's instructions. Purified DNA was eluted in a final volume of 50 µl and evaluated by agarose (0.8%) gel electrophoresis. The bands were then viewed by ethidium bromide staining. The concentration and purity of the phage DNA extracted was determined by a NanoDrop ND-1000 spectrophotometer (NanoDrop Technologies. Thermoscientifics. Wilmington. USA).

### Purification of bacterial DNA

Fifty ml of sewage and 100 ml of river water samples were passed through 0.45 µm polyvinylidene fluoride (PVDF) DURAPORE® membrane filters (Millipore, Bedford, Massachusetts), described by the manufacturer as low protein-binding membranes. These allowed the phages to pass through whilst bacteria were retained on the surface of the filter. To remove phages retained on the filters, 10 ml of PBS was added to the surface of the filter, gently agitated and removed by filtration. Two washing steps allowed high (99%) phage reduction without significant loss of bacteria [60]. The membrane containing retained bacteria was recovered in 4 ml of LB. The suspension was centrifuged at 3000 g for 10 min. To recover DNA from both Gram-positive and Gram-negative bacteria, the pellet was suspended in 180 µl of enzymatic solution (20 mg/ml lysozyme; 25 mg/ml lisostaphine, 20 mM Tris-HCl, pH = 8.0; 2 mM EDTA; 1,2% Triton) and incubated for 30 min at 37°C. DNA was then extracted using a QIAamp DNA Stool Mini Kit (Qiagen Inc., Valencia, USA), following the manufacturer's instructions.

### Transfection with antibiotic resistance genes

Twenty µl of phage DNA prepared as described above from three sewage samples (samples 6, 7 and 15) was transfected by electroporation into ap-sensitive, nal-resistant *E. coli* WG5 and C600nal^R^ strains (each culture containing 5×10^8^ CFU/ml). Electrocompetent cells were prepared and phage DNA was electroporated as described above and incubated for 2 h in LB at 37°C. The clones were selected on Chromocult plates supplemented with ap/nal. A 25% of the ap/nal-resistant colonies were randomly selected and screened for the presence of *bla*_TEM_ and *bla*_CTX-M_ genes with the corresponding primers for conventional PCR (Table 3). Positive amplification of the genes was confirmed by sequencing.

### Electron microscopy

The sewage and river samples were used as a source of bacteriophages. Viruses from the samples were partially purified by filtration and 100-fold concentrated (sewage) or 1000-fold concentrated (river water), by means of protein concentrators (100 kDa Amicon Ultra centrifugal filter units, Millipore, Bedford, MA), following the manufacturer's instructions. Ten-µl of each virus suspension was deposited on copper grids with carbon-coated Formvar films and stained with 2% KOH phosphotungstic acid (pH 7.2) for 2.0 min. Samples were examined in a JEOL JEM-1010 electron microscope operating at 80 kV.

### Sequencing and sequence analyses

The amplified DNA of each resistance gene cloned into the pGEM-T-Easy vector used to generate the standard was confirmed by sequencing. Amplicons of *bla*_TEM_, *bla*_CTX-M_ and *mec*A, generated by conventional PCR with primers described in Table 3, were electrophoretically analyzed in a 1% agarose gel, and bands were viewed by ethidium bromide staining. The bands were excised from the agarose gel and purified using a QIAquick Gel Extraction Kit (Qiagen Inc., Valencia, CA, USA), following the manufacturer's instructions. The purified amplicons were used as a template for sequencing. Sequencing was performed with an ABI PRISM Big Dye 3.1 Terminator Cycle Sequencing Ready Reaction Kit (Applied Biosystems, Spain) in an ABI PRISM 3730 DNA Analyzer (Applied Biosystems, Spain), following the manufacturer's instructions. All sequences were performed at least in duplicate.

Nucleotide sequence analysis searches for homologous DNA sequences in the EMBL and GenBank database libraries were carried out using Wisconsin Package Version 10.2, Genetics Computer Group (GCG), (Madison, WI). BLAST analyses were performed with the tools available on the National Institutes of Health (NIH) webpage: http://www.ncbi.nlm.nih.gov. Sequences were assembled with the MultAlin program available on the web page: http://bioinfo.genotoul.fr/multalin/multalin.html.

### Statistical analyses

Computation of data and statistical tests were performed using the Statistical Package for Social Science software (SPSS). One-way analysis of variance (ANOVA) tests were used to evaluate the differences between microbiological parameters in sewage and river samples and the differences between the resistance genes detected in bacterial and phage DNA. Evaluations were based on a 5% significance level in both cases (*P* 0.05). The box-plot graph used to compare the number of detected copies of the genes was done using EXCEL software (Microsoft® EXCEL 2000). The calculations performed to generate the box-plot graph included mean, standard deviation, media, quartiles and minimum and maximum values for each group of samples.
